# Self-Powered All-Inorganic Perovskite Photodetectors with Fast Response Speed

**DOI:** 10.1186/s11671-020-03460-4

**Published:** 2021-01-06

**Authors:** Ting Zhang, Shibin Li

**Affiliations:** School of Optoelectronic Science and Engineering, University of Electronic Science and Technology of China (UESTC), Chengdu, 610054 Sichuan China

**Keywords:** CsPbI_2_Br, CsPbIBr_2_, Anti-solvent processing, Self-powered capability

## Abstract

In this manuscript, the inorganic perovskite CsPbI_2_Br and CsPbIBr_2_ are investigated as photoactive materials that offer higher stability than the organometal trihalide perovskite materials. The fabrication methods allow anti-solvent processing the CsPbI_*x*_Br_3−*x*_ films, overcoming the poor film quality that always occur in a single-step solution process. The introduced diethyl ether in spin-coating process is demonstrated to be successful, and the effects of the anti-solvent on film quality are studied. The devices fabricated using the methods achieve high-performance, self-powered and the stabilized photodetectors show fast response speed. The results illustrate a great potential of all-inorganic CsPbI_*x*_Br_3−*x*_ perovskites in visible photodetection and provide an effective way to achieve high performance devices with self-powered capability.

## Introduction

Photodetectors (PDs), which can convert light into electrical signal, are important applications in image, optical communication, and environmental monitoring. Conventional PDs are mainly made by Si, ZnO, SiC, and HgCdTe, which either are expensive or require vacuum equipment to fabricate [1–4]. Most importantly, these commercial devices usually need precise and complex fabrication process which combines lithography, etching and deposition, limiting a wide deployment [5, 6]. Therefore, it is of great interest to develop new materials for high performance photodetector via facile fabrication method.

Recently, organometal trihalide perovskites (OTPs) have emerged as an attractive class of optoelectronic materials due to their outstanding optoelectronic properties, such as strong light absorption, high carrier mobility, low exciton binding energy, and low charge recombination rate [7–12]. These features make OTPs as the promising photovoltaic material candidates for next generation solar cells. Indeed, since the emergence of perovskite-based solar cells (PSCs) in 2009 [13], certified power conversion efficiencies (PCEs) of organic–inorganic halide PSCs have rapidly increased to 25.2% [14]. Besides, OTPs have shown great potentials in PDs [15–17], light emitting diodes (LEDs) [18–20], and lasers [21–24]. Although continuous progress have been made in improving the efficiency, some opto-electronic devices based on OTPs still face a bottleneck of stability problem [25, 26]. Due to the degradation and volatilization of organic groups, such as methylammonium (MA^+^) and formamidinium (FA^+^) cations, OTPs suffer an unsatisfactory long-term stability [26]. Previous reported works demonstrate all-inorganic perovskites (CsPbX_3_, X = I, Br, Cl) could solve the stability issue probably because of their intrinsic chemical stability [27–29]. Among these all-inorganic perovskites, black phase CsPbI_3_ has garnered great interest due to its suitable bandgap of 1.73 eV. Unfortunately, black-CsPbI_3_ is only stable at temperatures above 330 °C, which is not practical for applications [27]. Partially replacing iodide with bromide can stabilize the black phase of all-inorganic perovskites at room temperature and would not trade off the optical bandgap too much [30–32]. Recently, there are too many researches on CsPbI_*x*_Br_3−*x*_ perovskite solar cells, less works about PDs based on CsPbI_*x*_Br_3−*x*_ thin films have been reported. Moreover, the traditional PDs generally need external power sources to drive photogenerated carriers to input photocurrent. To meet the demands of next generated opto-electronic devices aimed at reduced weight, size and thickness, it is urgent to develop effective methods for the fabrication of PDs with self-powered capability.

Herein, we report high performance perovskite photodetectors based on solution-processed all-inorganic CsPbI_*x*_Br_3−*x*_ perovskite. At a low operation voltage of 2 V, the detectors showed broadband sensitivity covering visible-light spectrum and fast response speed down to 175 μs for CsPbI_2_Br PDs and 230 μs for CsPbIBr_2_ PDs. The detectivity and on/off ratio were calculated to be 10^11^ Jones and 10^3^, respectively. Even biased at 0 V, both the devices still worked well. This work provides a simple method to fabricate high-performance photodetectors in visible light with self-powered capability.

## Method

### Materials

Cesium iodide (CsI, 99.9%), lead iodide (PbI_2_, 99.99%), cesium bromide (CsBr, 99.99%) and lead bromide (PbBr_2_, 99.99%) were purchased from Xi’an Polymer Light Technology Corporation. Anhydrous dimethylformamide (DMF), dimethyl sulfoxide (DMSO) and diethyl ether (DE) were purchased from Sigma-Aldrich Corporation. Materials and solvents were used directly without purification.

The all-inorganic perovskite films were fabricated by one-step method using anti-solvent. First, to obtain the CsPbI_*x*_Br_3−*x*_ (*x* = 1, 2) precursor solution, stoichiometric-ratio PbI_2_, CsI, CsBr and PbBr_2_ were dissolved in a mixed solvent of DMF and DMSO (9:1 v/v) at 1.43 M and stirred for more than 2 h. All of the procedures should be operated in a nitrogen filled glovebox.

### Preparation

ITO-coated glass substrates were cleaned by acetone, ethyl alcohol and deionized water with each step for 15 min and dried in an oven. To form perovskite films, the precursors were spin-coated on pre-cleaned ITO substrates at a speed of 2000 rpm for 60 s, and dropped 500-μL antisolvent diethyl ether (DE, Sigma, 99.9%) at the last 20 s of the coating process. Then, the perovskite films were annealed at 65 °C for 5 min and 135 °C for 15 min. To compare the film-quality enhanced by anti-solvent DE, reference experiment, of which no antisolvent was introduced, also been conducted. Finally, 80-nm-thick interdigitated Au electrodes were thermally evaporated on perovskite films via mask.

### Measurements and Characterizations

The morphologies of as-prepared films were investigated by field emission scanning electron microscopy (FE-SEM). The phases and crystalline of as-synthesized inorganic perovskite were recorded by X-ray diffraction (XRD) patterns using an X-ray diffractometer (Cu Kα radiation, *λ* = 1.54056 Å). The UV–Vis absorption and PL spectra were performed using a UV–Vis spectrophotometer (Shimadzu UV-3101 PC) and a Hitachi F-4600 fluorescence spectrometer (Edinburgh, FLSP920) with an exciting wavelength of 410 nm, respectively. The current–voltage (I–V) curves were recorded by a Keithley 4200 Semiconductor Parametric Analyzer under the illumination of a LD light source (520 nm). The incident light intensity was measured by a commercial power meter with the type of Thorlabs PM 100D. Photocurrent and response speed were measured with an oscilloscope (Agilent DOS5012A) and an optical chopper modulating the light illuminated on the device. All the measurements were conducted in air atmosphere at room temperature.

## Results and Discussion

**Fig. 1:**
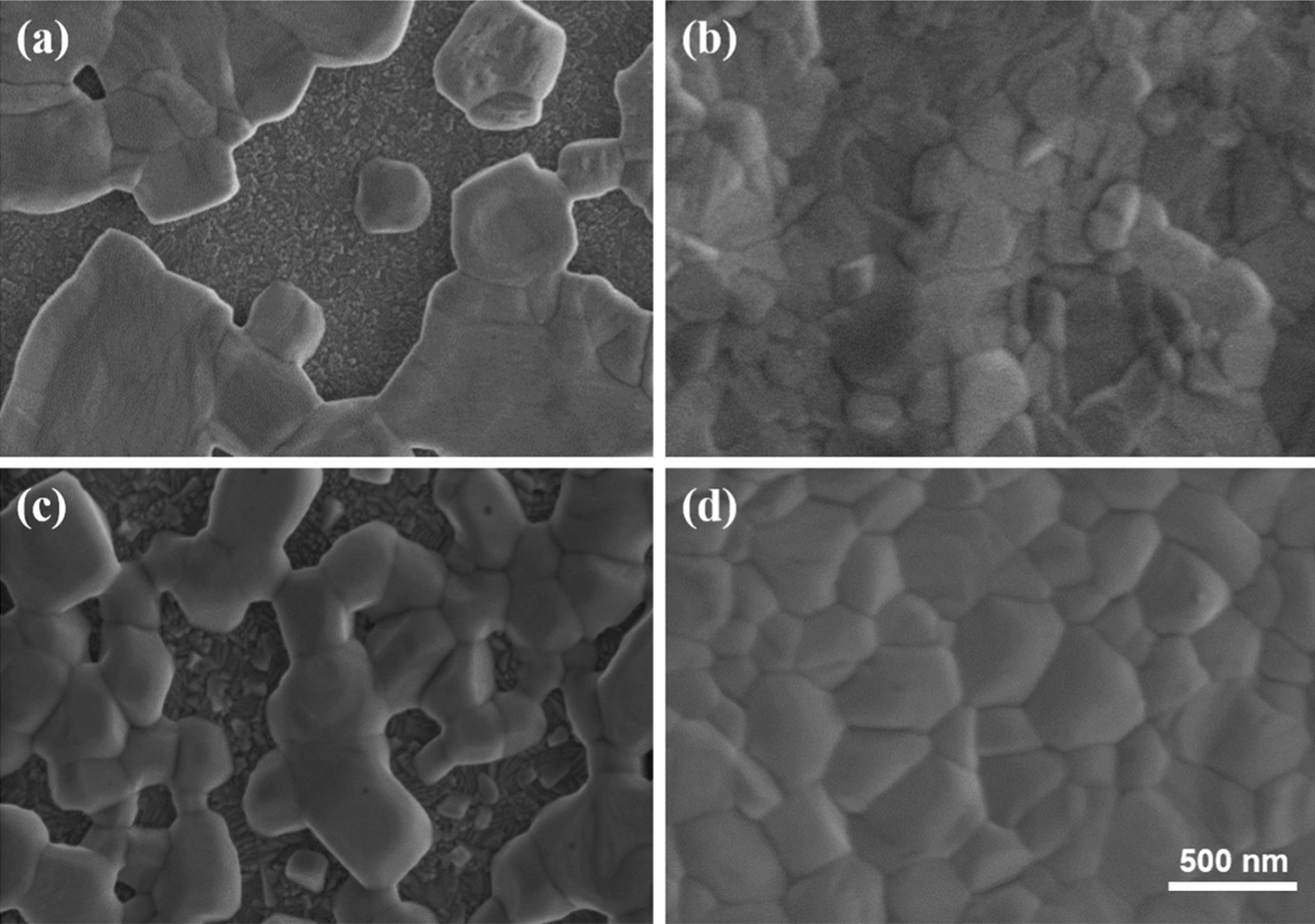
Top-view SEM images of the all-inorganic perovskite films. CsPbI_2_Br film **a** without **b** with DE treatment; CsPbIBr_2_ films **c** without **d** with DE treatment

**Fig. 2:**
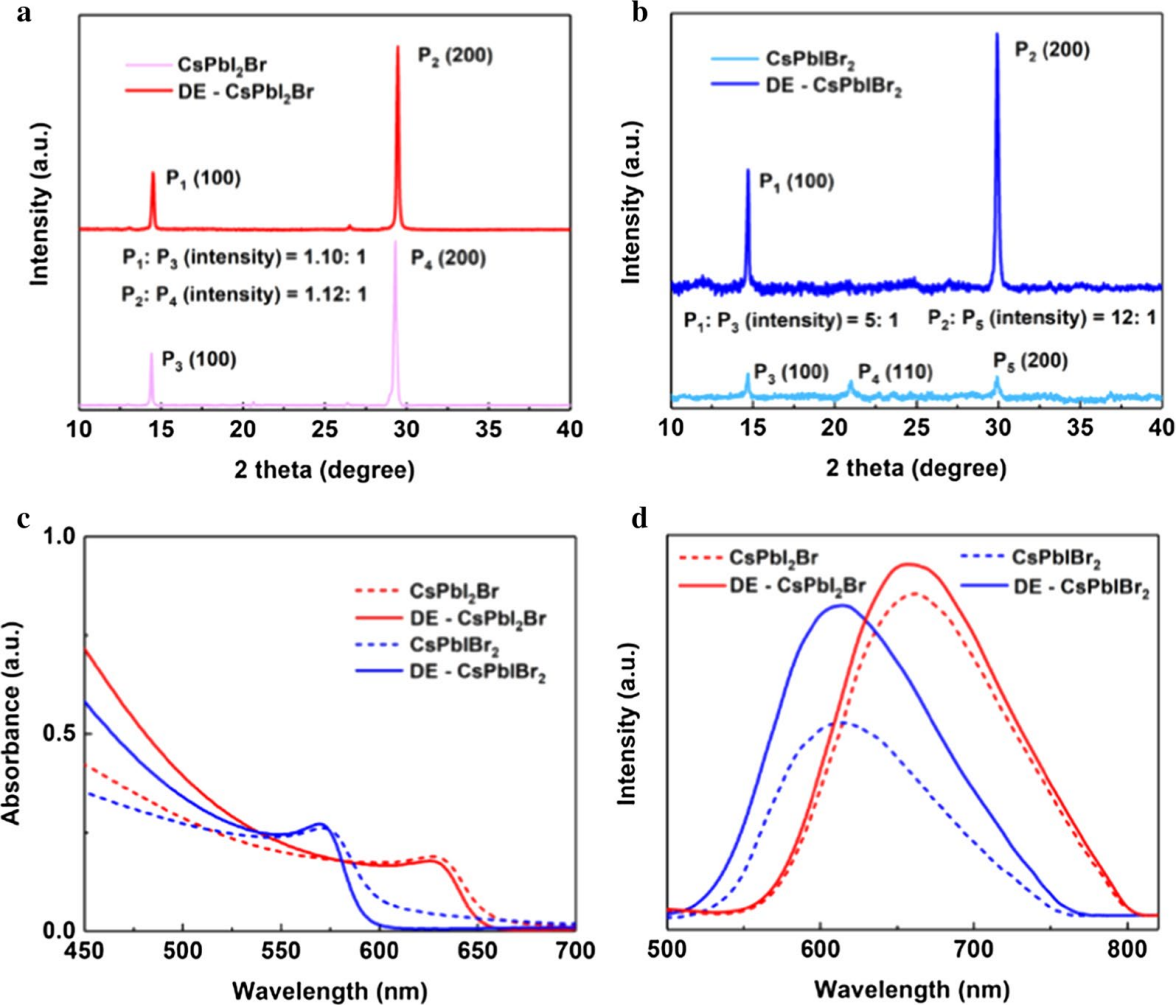
Comparison of **a** XRD patterns of CsPbI_2_Br films, **b** XRD patterns of CsPbIBr_2,_ **c** absorption of CsPbI_*x*_Br_3−*x*_, **d** photoluminescence spectra of CsPbI_*x*_Br_3−*x*_ with or without DE treatment

Figure 1 shows the top-view SEM images of CsPbI_2_Br and CsPbIBr_2_ thin films with or without DE treatment. Obviously, the pristine CsPbI_*x*_Br_3−*x*_ perovskite films are discontinuous and show large pinholes. After DE treatment, the film quality of CsPbI_*x*_Br_3−*x*_ is significantly enhanced showing higher coverage and compactness. To further investigate the crystal structure and phase purity of all-inorganic perovskite films, XRD patterns were recorded as displayed in Fig. 2a. For the pattern of CsPbI_2_Br film (as shown in Fig. 2b), the main peaks at 14.6° and 29.6° are assigned to the (100) and (200) crystallographic planes of the CsPbI_2_Br cubic perovskite structure, respectively. For the case of CsPbIBr_2_ film, the three peaks centered at 14.9°, 21.08° and 29.96° are associated with the (100), (110) and (220) planes of the CsPbIBr_2_ perovskite orthorhombic phase, respectively. In addition, the ratios of diffraction peak (P) 14.6° and 29.6° are calculated to be 1.10 and 1.12 for CsPbI_2_Br after DE treatment, respectively. This indicates that the CsPbI_2_Br perovskite film grow preferentially with (200) facet on DE treatment. Meanwhile, for the case of CsPbIBr_2_ perovskite film after DE treatment, the ratios of diffraction peak (P) 14.9° and 29.96° are calculated to be 5 and 12, respectively, which demonstrates the CsPbIBr_2_ perovskite film grow preferentially with (200) facet on DE treatment. Both the XRD results demonstrate that the DE treatment can improve the crystalline quality and phase purity of CsPbI_*x*_Br_3−*x*_ films obviously.

**Fig. 3:**
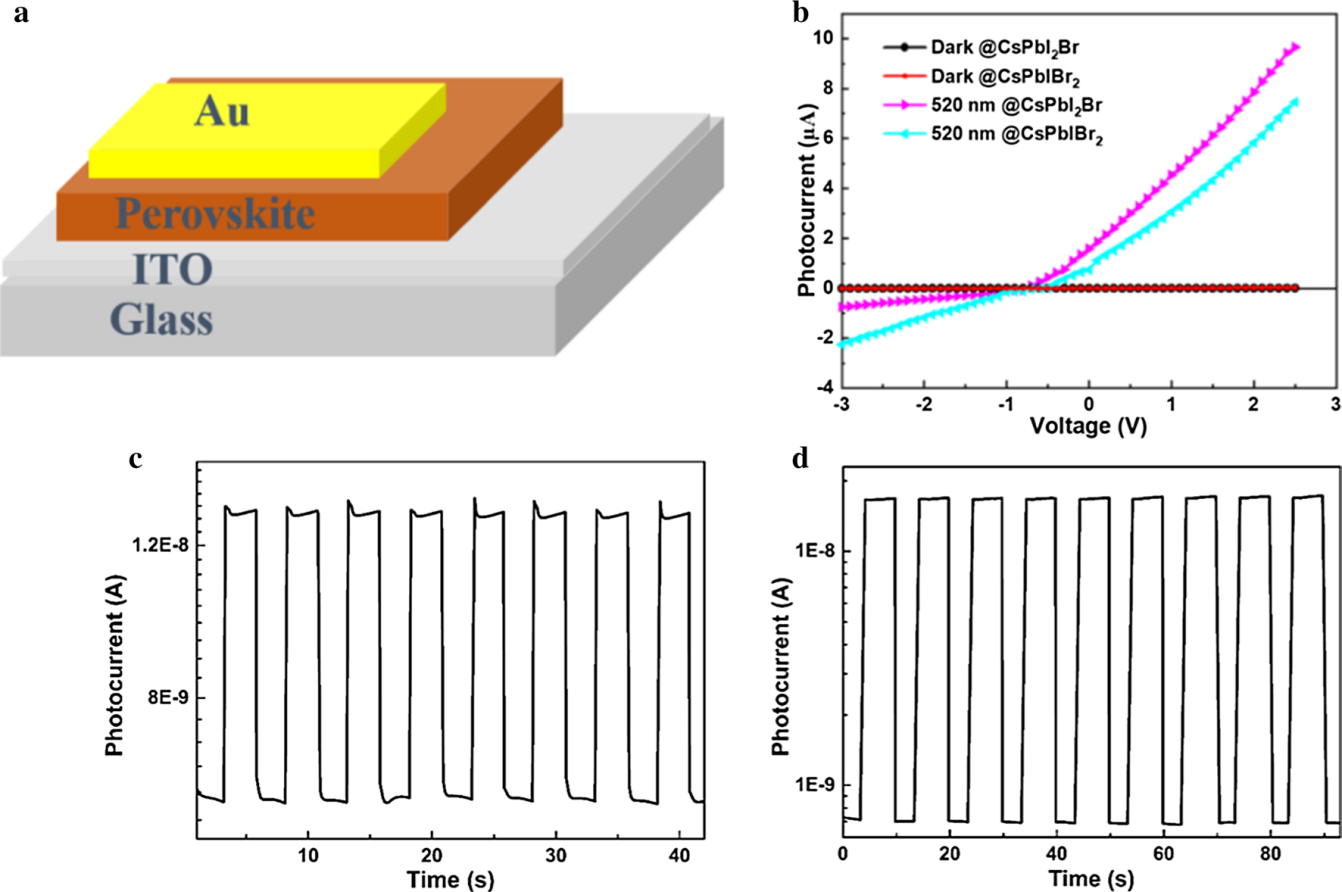
Optoelectronic performance of CsPbI_*x*_Br_3−*x*_ perovskite PDs. **a** schematic illustration of the CsPbI_*x*_Br_3−*x*_ perovskite photodetector, **b** current–voltage characteristics of the CsPbI_*x*_Br_3−*x*_ perovskite PDs in the dark and under 520 nm illumination with light intensity of 3.5 mW/cm^2^_,_ **c** temporal photoresponse of the CsPbI_2_Br PDs under 520 nm irradiation when biased at 0 V, **d** I–t curve of the CsPbI_2_Br PDs under 520 nm irradiation at 0 V

Furthermore, the optical properties of CsPbI_*x*_Br_3−*x*_ films with or without DE treatment were measured by UV–Vis absorption and PL spectrum. As shown in Fig. 2c, both CsPbI_2_Br and CsPbIBr_2_ samples present an improved absorbance after DE treatment. The absorbance spectra suggest these CsPbI_*x*_Br_3−*x*_ films can be used as active layers for visible photodetection effectively. Figure 2d is the PL spectra of CsPbI_2_Br and CsPbIBr_2_ films deposited on glass substrates. The PL peak of CsPbI_2_Br and CsPbIBr_2_ films located at 655 nm and 603 nm, respectively, which were in agreement with the previous reports [31]. For the cases treated by DE, the PL intensities increase significantly compared to those of untreated perovskite films. The increased PL intensities relate to the decreased trap density which would facilitate carriers in the excited state recombination to the ground radiatively. The results indicate that introducing the DE anti-solvent is an effective way to achieve better film quality and reduction in trap density in all-inorganic perovskite films. Therefore, we used the modified perovskite films as photoactive layers to fabricate all-inorganic CsPbI_*x*_Br_3−*x*_ perovskite PDs, with the structure shown in Fig. 3a.

Figure 3b shows the I–V curves of the devices in dark and under 520 nm light illumination. Under the illumination of 520 nm light source, the photocurrents increase greatly due to the large contribution from the photogenerated carriers. Obviously, the photocurrent curves of two different PDs show a rectification behavior, indicating that junction barriers exist between the ITO and perovskite films. These junction barriers could be ascribed to Schottky contact formed at the ITO/CsPbI_2_Br or ITO/CsPbIBr_2_ interfaces and the surface states, such as surface defects, vacancies and absorption [33]. The phenomenon always exists in previously reported perovskite PDs [34–36]. When the device was biased at 0.1 V, the detector based on CsPbI_2_Br perovskite showed a dark current of ~ 2 nA. Once exposed to a 520 nm laser diode (LD) light source with the illumination intensity of 3.5 mW/cm^2^, the photocurrent increased to μA, achieving a high on/off ratio larger than 10^3^. For the case of CsPbIBr_2_ photodetector biased at 0.1 V, the dark current was 2.45 nA, which resulted in an on/off ratio of 10^3^ as well. When the light source was switched on and off, both the devices showed rapid response in the current–time (I–t) curves at zero bias, as displayed in Fig. 3c, d. In addition, from Fig. 2b, the values of open-circuit voltage of CsPbI_2_Br and CsPbIBr_2_ photodetectors are − 0.74 and − 0.68 V, respectively. When light was on, the photocurrent increased sharply and then decreased rapidly once the light was turned off. It is noted that I–t curves were measured by controlling the LD light source to achieve on/off recycles. The results further illustrate that the CsPbI_*x*_Br_3−*x*_ perovskite photodetectors show a good light-switching behavior and reproducible photocurrent response to periodic on/off light. In addition, the I–t curves fit well with the I–V curves, further indicating the devices have fast response speed and lower delaying properties. As the critical parameters for evaluating a commercial photodetector, responsivity (*R*) and specific detectivity (*D*) are analyzed. When the dark current is assumed to be dominated by shot noise, *D* can be calculated by the following equation

$$D* = \frac{{J_{{{\text{ph}}}} }}{{L_{{{\text{light}}}} }}\frac{1}{{(2qJ_{{\text{d}}} )^{{{\raise0.7ex\hbox{$1$} \!\mathord{\left/ {\vphantom {1 2}}\right.\kern-\nulldelimiterspace} \!\lower0.7ex\hbox{$2$}}}} }} = \frac{R}{{(2qJ_{{\text{d}}} )^{{{\raise0.7ex\hbox{$1$} \!\mathord{\left/ {\vphantom {1 2}}\right.\kern-\nulldelimiterspace} \!\lower0.7ex\hbox{$2$}}}} }}$$

where $J_{{\text{d}}}$ is the dark current, $J_{{{\text{ph}}}}$ is the photocurrent, $L_{{{\text{light}}}}$ is the incident light intensity. *R* means the photocurrent generated per unit intensity of the incident light, which reflects the efficiency of the detector responds to the incident light signals.

**Fig. 4:**
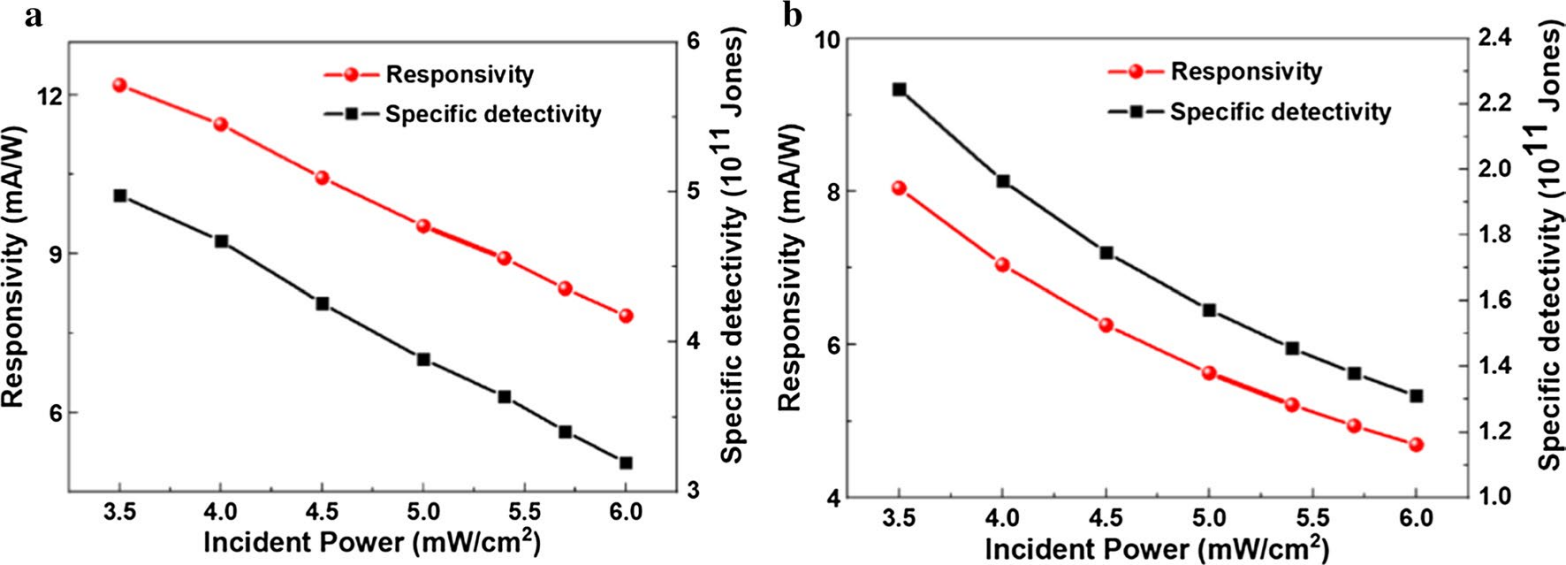
Responsivity and specific detectivity of CsPbI_*x*_Br_3−*x*_ perovskite PDs. **a** CsPbI_2_Br perovskite photodetector, **b** CsPbIBr_2_ perovskite photodetector

Figure 4a, b shows the detectivity and responsivity values of CsPbI_2_Br and CsPbIBr_2_ perovskite photodetectors measured at different incident light power. For CsPbI_2_Br device, under weak (3.5 mW/cm^2^) and strong (6 mW/cm^2^) illumination, D^*^ were calculated to be 4.9 × 10^11^ and 3.2 × 10^11^ Jones (${\text{Jones}} = {\text{cm}} \times {\text{Hz}}^{\frac{1}{2}} \times {\text{W}}^{ - 1}$), respectively. For the case of CsPbIBr_2_ photodetector, D^*^ under weak and strong light illumination were ~ 2.3 × 10^11^ and 1.3 × 10^11^ Jones, respectively. The calculated D^*^ and *R* values decreased linearly with the increase in incident light intensity. Under a strong illumination (6 mW/cm^2^), the CsPbI_2_Br and CsPbIBr_2_ detectors showed *R* values of 8 and 4.6 mA/W, respectively. Under a weak illumination (3.5 mW/cm^2^), both the above-mentioned PDs showed good performance with *R* of 12 and 8 mA/W, respectively. The high detectivity means the weak light signals could also be detected and transferred into large photocurrent. This is attributed to the improved all-inorganic perovskite film quality via DE treatment.

**Fig. 5:**
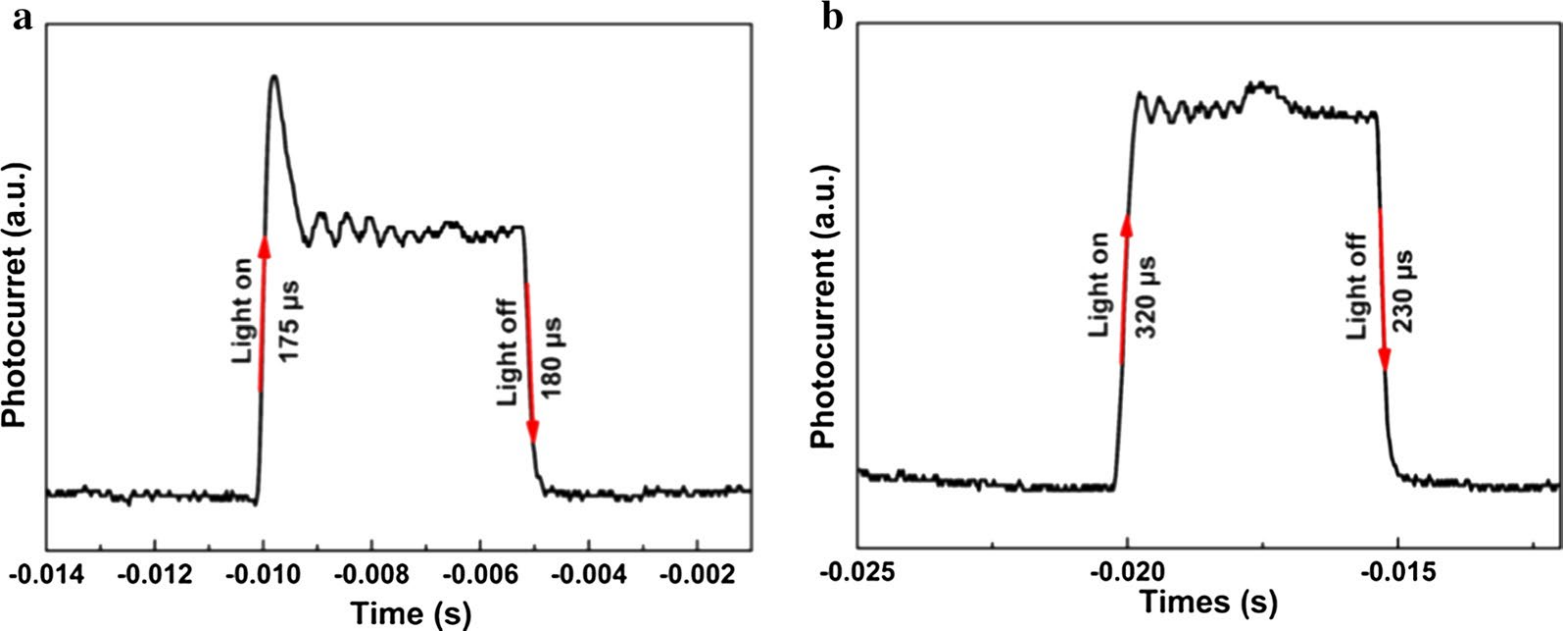
Response speed of CsPbI_*x*_Br_3−*x*_ perovskite PDs. **a** CsPbI_2_Br perovskite photodetector, **b** CsPbIBr_2_ perovskite photodetector

Further, the response speed is a figure-of-merit for photodetectors to characterize the device. We defined the rise time as the time spent on rising from 10 to 90% of maximum photocurrent, and vice versa means the decay time. To obtain the detailed response speed, an oscilloscope was used to control and record the temporal response. As plotted in Fig. 5a, b, the rise time and decay time for CsPbI_2_Br device were extracted to be 175 and 180 μs, respectively. Meanwhile, the rise and decay time for CsPbIBr_2_ were 320 and 230 μs, respectively. The fast response time means less electronic trap states exist at the interface of perovskite/metal, which could affect the charge transport and collection.

## Conclusion

In summary, we reported a facile fabrication of self-powered all-inorganic CsPbI_*x*_Br_3−*x*_ PDs with fast response speed. Under 520 nm laser illumination with 3.5 mW/cm^2^, the CsPbI_2_Br devices showed a responsivity up to 12 mA/W, a detectivity values of 10^11^ Jones and on/off ratios larger than 10^3^. And the CsPbIBr_2_ devices showed a responsivity values of 8 mA/W and detectivity up to 10^11^ Jones. The devices can work well even at zero bias. This work inspires the development of all-inorganic perovskite for solution-processed, self-powered and high-performance photodetectors.

## Data Availability

The data generated or analyzed during the current study are obtained from the corresponding author on reasonable request.

## Abbreviations

PDs: Photodetectors
OTPs: Organometal trihalide perovskites
DE: Diethyl ether
DMF: Dimethylformamide
DMSO: Dimethyl sulfoxide
SEM: Scanning electron microscope
UV–Vis: Ultraviolet–visible
XRD: X-ray diffraction
